# Different TP53 mutants in p53 overexpressed epithelial ovarian carcinoma can be associated both with altered and unaltered glycolytic and apoptotic profiles

**DOI:** 10.1186/s12935-018-0514-2

**Published:** 2018-01-30

**Authors:** Stephanie Antoun, David Atallah, Roula Tahtouh, Nada Alaaeddine, Malak Moubarak, Abir Khaddage, Eliane Nasr Ayoub, George Chahine, George Hilal

**Affiliations:** 1 Cancer and Metabolism Laboratory, Faculty of Medicine, Saint Joseph University, Damascus Road, Riad el Solh, Beirut, 1107 2180 Lebanon; 2 Obstetrics and Gynecology Department, Hotel-Dieu De France Hospital, Beirut, Lebanon; 3 Regenerative Medicine and Inflammation Laboratory, Faculty of Medicine, Saint Joseph University, Beirut, Lebanon; 4 Anatomy and Pathology Department, Hotel-Dieu De France Hospital, Beirut, Lebanon; 5 Anesthesiology Department, Hotel-Dieu De France Hospital, Beirut, Lebanon; 6 Oncology Department, Hotel-Dieu De France Hospital, Beirut, Lebanon

**Keywords:** Apoptosis, Glucose metabolism, Mutation, p53 overexpression, Ovarian cancer

## Abstract

**Background:**

p53 is a tumor suppressor and key regulator of glycolysis in cancer cells, however highly mutated in tumors. In ovarian cancer, studies concerning p53 mutations focus on the DNA binding domain since the majority of hotspot mutations affects this region. Yet, mutations in other regions such as the proline rich domain may also affect the protein’s expression and activity. The aim of this study is to investigate the effect of various positions of mutations in TP53 gene on glycolysis, apoptosis and transcription of p53 target genes.

**Methods:**

Mutations frequency and their effect on p53 expression were assessed by PCR-SSCP, sequencing and immunohistochemistry on 30 ovarian cancer biopsies. Six tumors were cultured, as well as SK-OV-3, OVCAR-3 and Igrov-1. SK-OV-3 cells were transfected with 2 TP53 mutants. p53 transcriptional activity was assayed by qPCR, apoptosis by flow cytometry and glycolysis by glucose and lactate measurements, with quantification of glycolytic enzymes expression.

**Results:**

Our results showed a high frequency of the P72R mutant, associated with p53 overexpression in the ovarian biopsies. However, P72R mutant cells showed similar apoptosis and glycolysis as WT cells. DNA binding domain mutations decreased the transcriptional activity of the protein and increased glucose consumption and lactate production.

**Conclusion:**

Despite the overexpression of the P72R mutated protein in the biopsies, it showed a similar apoptotic activity and glucose regulation ability as WT p53. Knowing that p53 expression status is used for chemotherapeutic approaches and prognosis in ovarian cancer, the results obtained highlight the importance of locating TP53 mutations.

**Electronic supplementary material:**

The online version of this article (10.1186/s12935-018-0514-2) contains supplementary material, which is available to authorized users.

## Background

Ovarian cancer has the highest mortality rate among gynecological malignancies in the Western world [1]. The Cancer Genome Atlas Research Network demonstrates that high-grade serous cancer is characterized by TP53 mutations in up to 96% of the cases [2]. In addition, P53 is more highly expressed in high-grade tumors, in cancers with metastasis as well as in younger patients [3].

The p53 protein is a 393 amino acid phosphoprotein with five main active domains, with specific functions linked to each one of these domains [4]. This protein enhances the transcription of its target genes, such as p21, a kinase inhibitor implicated in cell cycle arrest and apoptosis initiation [5], and MDM2, which is responsible of the repression and degradation of p53 [6].

Various types of TP53 mutations are observed in 40–80% of epithelial ovarian cancers, contributing either to an inactive or a truncated p53 protein [7]. The majority of mutations affecting TP53 are usually located in exons 5–8, coding for the DNA binding domain of the protein. This domain plays a key role in the activation of the transcription of p53 target genes [8]. Single point mutation in this area leads to the formation of a complete protein with a decreased affinity to its binding sites on its target genes. In addition to the DNA binding domain, the core domain of this protein contains also a proline rich region, which regulates the ability of the protein to induce apoptosis. This region of the gene coded by exon 4 is also prone to mutations. Actually, a single nucleotide polymorphism (SNP), affecting codon 72 of exon 4, leads to the conversion of proline (CCC) to arginine (CGC) [9]. Recent studies demonstrated an association between this polymorphism and the risk of cancer development, along with a variation in patient’s response to chemotherapy [10].

In addition to its role in proliferation control, senescence and apoptosis, p53 regulates glycolysis and oxidative phosphorylation by inhibiting the first and activating the second [11, 12]. One of these genes is TIGAR (TP53-inducible glycolysis and apoptosis regulator), an inhibitor of phosphofructokinase, which is responsible of the hydrolysis of fructose-2,6-bisphosphate and fructose-1,6-bisphosphate [13]. In opposition to TIGAR, the glucose transporters GLUT-1, GLUT-3 and GLUT-4 are repressed by p53, which leads to a decrease in glucose absorption [14]. Mutations affecting the DNA binding domain of TP53 decrease the ability of p53 to regulate the expression of glucose transporters, which increases glucose metabolism and energetic supply [15]. p53 also represses the transcription of pyruvate kinase (PK), phosphofructokinase (PFK) and aldolase and enhances the expression of pyruvate dehydrogenase (PDH) [16]. In addition, p53 modulates glucose metabolism via its direct effect on the key enzyme of the pentose phosphate pathway, the glucose-6-phosphate dehydrogenase (G6PD) [17].

In the present study, we demonstrated that p53 functions are differently altered depending on the site of the mutation affecting the gene. In fact, alterations affecting both proline rich domain and DNA binding domain lead to p53 overexpression. However, P72R polymorphism doesn’t affect the protein’s transcriptional activity, apoptosis induction or glycolysis regulation. In the contrary, mutations in the DNA binding domain contribute to a more pronounced decrease in glucose metabolism.

## Materials and methods

### Patient population and data collection

The study population included 30 Lebanese patients suffering from epithelial ovarian carcinoma who had undergone debulking surgery at the Hotel-Dieu de France hospital between 2014 and 2017. Fresh tissue was collected after surgery, along with all histopathological and clinical data, including the age of the patient, date of surgery, histological subtype, stage and grade, and follow-up information. Informed consent from each patient was obtained at the time of the surgery, according to the regulations of the institution. The ethical permission for sample collection and manipulation was obtained from the ethical committees of both Saint Joseph University and Hotel-Dieu de France hospital.

### Immunohistochemistry for p53

Fresh tissue samples were paraffin embedded, sectioned at 4 μm, mounted on slides and fixed for 10 min in acetone. The tissue sections were then incubated with a primary mouse monoclonal p53 antibody (DO-7, 1 μg/mL, 1/100, BioGenex Fermont CA), followed by incubation with a secondary rabbit anti-mouse IgG antibody and a tertiary mouse peroxidase anti-peroxidase antibody. Staining of the initial tissue section with H&E was performed to confirm the histopathological type of the ovarian tumor. One pathologist (A.K.) reviewed the slides, and tumors with 40% or more stained nuclei were considered p53 overexpressing.

### Cell extraction and culture

Ovarian cancer samples were examined macroscopically, and carcinomatosis was separated from normal tissue according to the aspect of the tissue sections. These carcinomatosis were cut into small pieces and were incubated with collagenase IV (Sigma Chemical Co., St. Louis, MO, USA) for 2 h in a 37 °C shaker incubator. The digest, containing ovarian cancer cells, was harvested and cultured in DMEM (4.5 g/L glucose) supplemented with 10% fetal bovine serum (FBS) and 1% penicillin/streptomycin (PS) (Sigma Chemical Co., St. Louis, MO, USA). As for the cell lines, the Sk-Ov-3 and Igrov-1 cell lines were purchased from the American Type Cell Culture (ATCC, USA) and were cultured in 4.5 g/L DMEM + 10% FBS + 1% PS as recommended. The Ovcar-3 cell line was also purchased from the ATCC and was cultured in F12 medium with 20% FBS and 1% PS. All the cells were incubated in a humidified incubator at 37 °C with 5% CO_2_.

### DNA purification and PCR-SSCP

DNA was extracted from the tissue section using the NucleoSpin Tissue for DNA, RNA and protein purification kit (Macherey–Nagel, USA) according to the manufacturer’s protocol. The amplification of each of the 10 exons was performed by PCR using the specific primers listed in Additional file 1: Table S1. The oligonucleotide primers were designed to span the entire exon and a sufficient flanking intron sequence to include the splice junction mutations for the analysis. SSCP was performed as follows: 100 ng of DNA was amplified for 35 cycles using the 5× FIREPol PCR mastermix ready to load (SOLIS BIODYNE, Estonia). The PCR product was denatured by heat shock and was run on a 12% polyacrylamide gel at 160 V for 2 h. The gel was stained by SYBR gold (Qiagen Inc.) for 15 min and visualized using the UVP BioImaging system. The wild type p53 gene in the MCF-7 cell line was used as a control. All the samples that showed altered mobility were further characterized by DNA sequencing for the altered exon.

### DNA sequence analysis for p53

The altered exons identified by SSCP were assessed by DNA sequencing. DNA was reamplified with the suitable primer, and the PCR product was purified using the PCR purification kit (Qiagen, Inc). The purified strands were analyzed using the Applied Biosystems 3500 and the BigDye terminator 1.1 according to the manufacturer’s protocol. The ovarian carcinoma cases that showed no alteration in motility by the SSCP technique but exhibited an overexpression of p53 by immunohistochemistry were subjected to complete DNA sequencing for exons 2–11.

### Plasmid construction and transfection

The Sk-Ov-3 cell line was transfected with 4 different types of plasmids, including pBABE-neo, Flag-p53/pRK5 and pCMV-Neo-Bam p53 R249S plasmids, which were purchased from AddGene (USA), and a fourth plasmid was constructed using the GeneArt Site-Directed Mutagenesis PLUS kit (Invitrogen, life technologies) according to the manufacturer’s protocol. The Flag-p53/pRK5 plasmid was used as a template, and a 25 bp set of primers (Additional file 1: Table S1) was designed containing the point mutation in the middle.

### RNA purification and quantitative real time PCR

The cultured cells that were extracted from the biopsies, the cells from the three cell lines and the transfected Sk-Ov-3 cells were subjected to RNA extraction using the Nucleospin RNA extraction kit (Macherey–Nagel, USA) according to the manufacturer’s protocol. cDNA synthesis was performed with 40 ng of RNA using the iScript cDNA Synthesis kit (Bio-Rad, USA). Genes expression was quantified using the QuantiFast SYBR Green PCR Kit (Qiagen, Inc.) by qPCR according to the manufacturer’s protocol. Β actin and GAPDH were used as internal controls. Adequate primers for all the genes are listed in Additional file 1: Table S1.

### Measurement of glucose consumption and lactate production

The levels of glucose absorption by the ovarian cultured biopsies, the cell lines and the transfected cells were evaluated using the glucose oxidase from *Aspergillus niger* kit (Sigma Chemical Co., St. Louis, MO, USA) following the manufacturer’s protocol. Lactate production was evaluated in the same supernatant samples using the PAP Lactate kit (BioMerieux, France) according to the kit’s protocol. The results were normalized according to the cell count.

### Cell apoptosis-Annexin V affinity assay

The apoptosis levels of the transfected Sk-Ov-3 cells were determined using the Annexin V-FITC Apoptosis Detection Kit (Abcam) as recommended by the manufacturer. Briefly, the cells were transfected with the four types of vectors for 24 and 48 h. The transfected cells were then labeled with Annexin V-FITC and detected by flow cytometry.

#### Proliferation assay

The cell viability was determined using the 3-(4,5-dimethylthiazol-2-yl)-5-(3-carboxymethoxyphenyl)-2-(4-sulfophenyl)-2*H*-tetrazolium salt assay as reported by the manufacturer’s instructions (Takara Bio Inc, Ostu, Shiga, Japan).

### Statistical methods

The numerical values are reported using the mean ± SD. P-values were calculated by the means of an unpaired Student’s *t*-test using the GraphPad QuickCalcs online software (http://www.graphpad.com/quickcalcs/ttest1.cfm). P < 0.05 were considered statistically significant.

## Results

### P53 mutations and protein overexpression

A total of 30 ovarian cancer biopsies were included in the final study. A summary of the clinical and histopathological analysis is listed in Additional file 2: Table S2. In the 30 biopsies, we found a total of 17 sequence alterations in the TP53 gene, detected in 13 cases, using PCR-SSCP and direct sequencing techniques. Nine of these cases presented one mutation, and the other 4 showed 2 mutations affecting the gene (Table 1). Six intronic mutations were detected, mainly affecting introns 2 and 7, and 11 mutations were exonic, mainly located on exons 3, 4, 7 and 9. In addition, 69% of the mutations were restricted to the proline rich domain responsible of apoptosis activation (9 patients out of 13), 23% were restricted to the core domain (3 out of 13), and 8% were localized in both active sites (1 case out of 13). Two substitutions P72R at exon 4 and R282W at exon 7 are known single nucleotide polymorphisms (SNPs), whereas the rest of them are non-frequent mutations. The substitution of proline by arginine at codon 72 was detected in six of the ovarian cancer cases (patients number 4, 6, 7, 11, 15 and 25), along with an overexpression at the protein’s level as detected by immunohistochemistry (Table 1). In fact, immunohistochemistry results showed a 100% correlation between p53 mutations and overexpression of the protein, which demonstrates again that IHC is a proxy for mutant protein detection. Four patients (tumors 9, 11, 14 and 18) showed negative results using the PCR-SSCP technique, but mutations were detected by direct sequencing.

**Table 1:** p53 mutations and protein overexpression in ovarian carcinoma

Tumor	SSCP results	Exon/intron	Type	Nucleotide	AA change	IHC results
2	+	Intron 2	Substitution	15902 A<G		++
3	+ +	Exon 3 Intron 2	Substitution Substitution	16147 G<A 15963 A<T	L<K	+++
4	+	Exon 4	Substitution	16397 C<G	P<R	++
5	+ +	Exon 4 Exon 7	Substitution Substitution	16494 G<T 18330 C<T	G<V R<W	+++
6	+	Exon 4	Substitution	16397 C<G	P<R	+++
7	+ −	Exon 4 Intron 2	Substitution Substitution	16397 C<G 15854 G<C	P<R	++
9	− −	Exon 7 Exon 9	Substitution Deletion	18429 T<G 19001	A<E	++
11	− −	Exon 4 Intron 7	Substitution Substitution	16397 C<G 18536 T<C	P<R	++
14	−	Intron 2	Substitution	15896 T<C		++
15	+ −	Exon 3 Exon 4	Substitution Substitution	16147 G–A 16397 C<G	L<K P<R	+++
18	−	Intron 7	Substitution	18603 A<T		++
25	+	Exon 4	Substitution	16397 C<G	P<R	+++
29	+	Intron 7	Substitution	18549 C<G		+++

### Effect of mutations on p53 expression and transcriptional activity

**Fig. 1:**
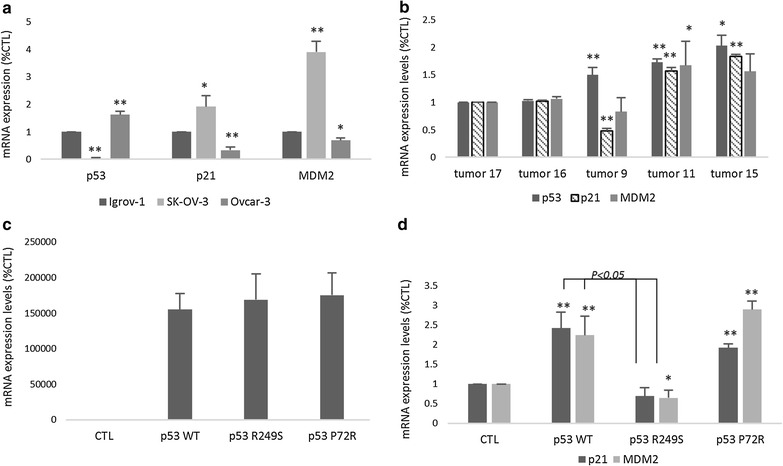
Effect of the mutation sites on p53 expression and transcriptional activity. According to the mutation analysis, five tumors were cultured in 4.5 g/L DMEM medium (**b**), with the 3 ovarian cancer cell lines, including IGROV-1, SK-OV-3 and OVCAR-3 (**a**). P53-null SK-OV-3 cells were transfected with four plasmids for 48 h using Attractene transfection reagent (**c**, **d**). RNA was extracted from all the cells listed above, and the quantification of the expression of p53, p21 and MDM2 was performed by qPCR using the SYBR green mix. Each value represents the mean of three assays. The control was set to 1, and the values were compared to the control. The results are displayed as the mean ± SD. *P < 0.05 versus the control, and **P < 0.01 versus the control

The expression levels of p53 target genes, p21 and MDM2, were evaluated in 3 ovarian cancer cell lines (IGROV-1 with WT p53, OVCAR-3 with exon 7 mutation in p53, and p53-null SK-OV-3), and 5 ovarian cancer cases (cases 16 and 17 with WT p53, case 9 with a DNA binding domain mutation and cases 11 and 15 with proline rich domain mutations). The results shown in Fig. 1 reveal the inhibitory effect of p53 DNA binding domain mutations on its transcriptional activity. In fact, p53 core domain mutations, in the OVCAR-3 cells and tumor 9, significantly decreased p21 and MDM2 genetic expression (Fig. 1a, b). An opposing effect was present when the mutation affected the proline rich domain of the gene. In fact, knowing that no mutations were detected in the DNA binding site, the overexpression of p53 in tumors 11 and 15 might be behind the significant overexpression of p21 and MDM2 (Fig. 1b).

To confirm the direct effect of the different domain mutations on p53 transcriptional activity and to exclude any external modulation of p21 and MDM2, p53 null SK-OV-3 cells were transfected with 4 different types of plasmids, including WT p53, mutant R249S p53, mutant P72R p53 and an empty vector as a negative control. The same effect was observed after the transfection of SK-OV-3 cells with both WT and P72R p53. In fact, p21 and MDM2 expression increased by 2-folds in the presence of both forms of p53 (*P* < *0.05).* This similarity observed between WTp53 cells and P72R p53 cells can be explained by the position of the mutation affecting the proline rich domain of the gene, and not the DNA binding domain responsible for the transcriptional activity of the protein. In contrast, no significant effect on p21 and MDM2 genetic expression was detected after the SK-OV-3 cells were transfected with p53 R249S (Fig. 1d). However, when cells transfected with R249S p53 are compared to WTp53 cells, a 1.5-fold decrease in p21 and MDM2 expression is observed (*P* < *0.05*).

### Effect of p53 mutations on cell proliferation and apoptosis

**Fig. 2:**
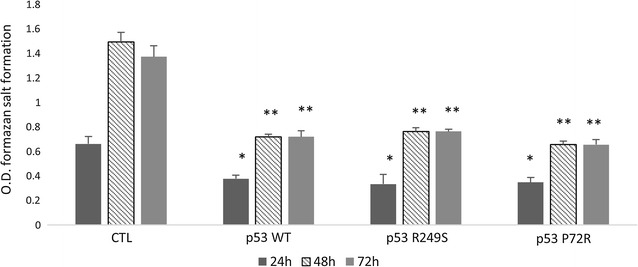
Effect of p53 domain mutations on cellular proliferation. The proliferation of the transfected SK-OV-3 cells was performed using the tetrazolium salt by measuring the ability of the mitochondrial enzymes to transform this salt into formazan. Briefly, transfection was done using Attractene for 24, 48 and 72 h. The formazan concentrations were measured at t = 0 min, and then, the cells were incubated with tetrazolium salt for 45 min, and the formazan concentration was measured using an ELISA reader at 450 nm wavelength. The results are expressed by the mean ± SD. Each condition was performed in triplicate. *P < 0.05 versus the control, and **P < 0.01 versus the control

**Fig. 3:**
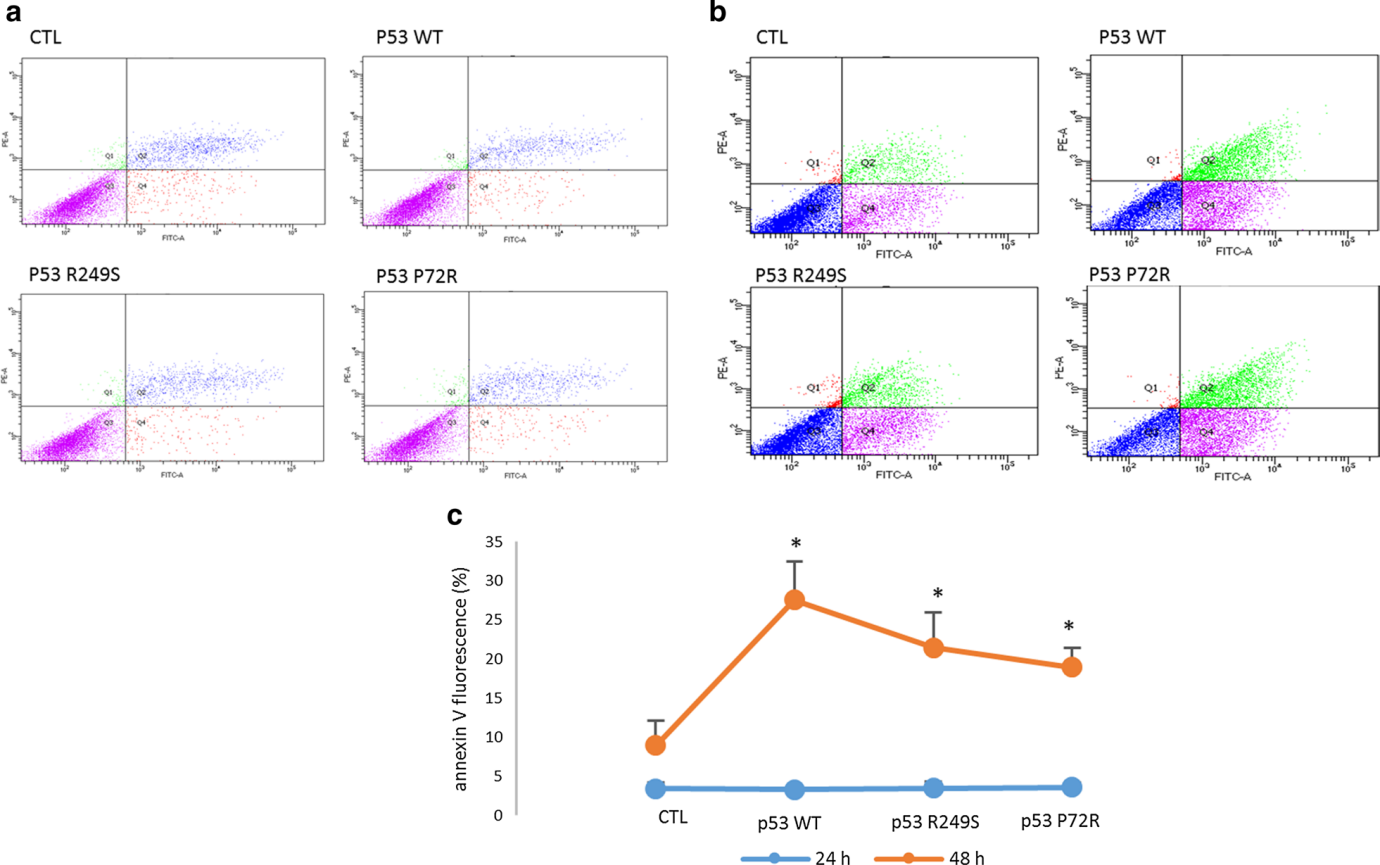
Effect of p53 site mutations on apoptosis initiation. The influence of the mutations affecting the various domains of the p53 gene on apoptosis was assessed in the transfected SK-OV-3 cells. After transfection for 24 and 48 h, the cells were harvested and Annexin V was performed by flow cytometry. **a**, **b** Are examples of the results obtained by flow cytometry. **a** Represents 24 h and **b** is 48 h after the transfection using the four plasmids (ctl, p53 WT, p53 R249S and p53 P72 R for 1, 2, 3 and 4, respectively). Each experiment was performed three times, and the mean ± SD are displayed in **c**. *P < 0.05 versus the control

Cell proliferation of transfected SK-OV-3 cells was assayed. The highest decrease was observed after transfection with the mutant p53 P72R (Fig. 2a, b). To study the outcome of the p53 mutations on apoptosis initiation, the transfected SK-OV-3 cells were screened using Annexin V by flow cytometry after a 24 and 48 h incubation. The results clearly showed an increase in the number of apoptotic cells when the cells were transfected with the all 3 vectors (Fig. 3).

### Effect of p53 mutations on glucose consumption and lactate production

**Fig. 4:**
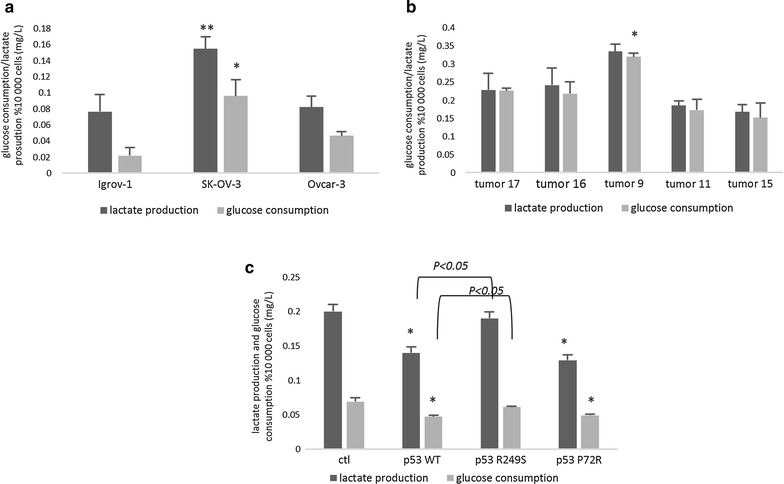
Effect of the different domain mutations on glucose consumption and lactate production. After a 48 h incubation in 4.5 g/L DMEM cell culture medium, the supernatants of the IGROV-1, SK-OV-3, and OVCAR-3 cells (**a**), the transfected cells (**c**) and the tumor cells (**b**) were collected. Glucose consumption levels were measured by the glucose oxidase test, while lactate production was assessed by PAP lactate. The values are presented as the mean ± SD of more than three experiments for each cell type and condition. *P < 0.05 versus the control, and **P < 0.01 versus the control

After glucose intake and lactate production assessment, The p53 null SK-OV-3 cell line exhibited a higher level of glucose absorption and lactate secretion compared to the WT IGROV-1 cells (Fig. 4a). An increase in these two parameters was detected in the tumor 9 cultured cells, exhibiting a DNA binding domain mutation, whereas tumors 11 and 15 showed no difference compared to the WT p53 tumors (Fig. 4b).

To prove that the p53 mutations were behind the alteration in glucose consumption and lactate formation, both concentrations were also measured in the transfected SK-OV-3 supernatants. The results in Fig. 4c clearly show the inhibitory effect of p53 when it is present in both the WT and mutant P72R forms. Interestingly, the mutant p53 R249S showed an increase in glucose assimilation and lactate secretion compared to the WT p53 (Fig. 4c).

### Effect of p53 mutations on glycolytic enzyme expression

**Fig. 5:**
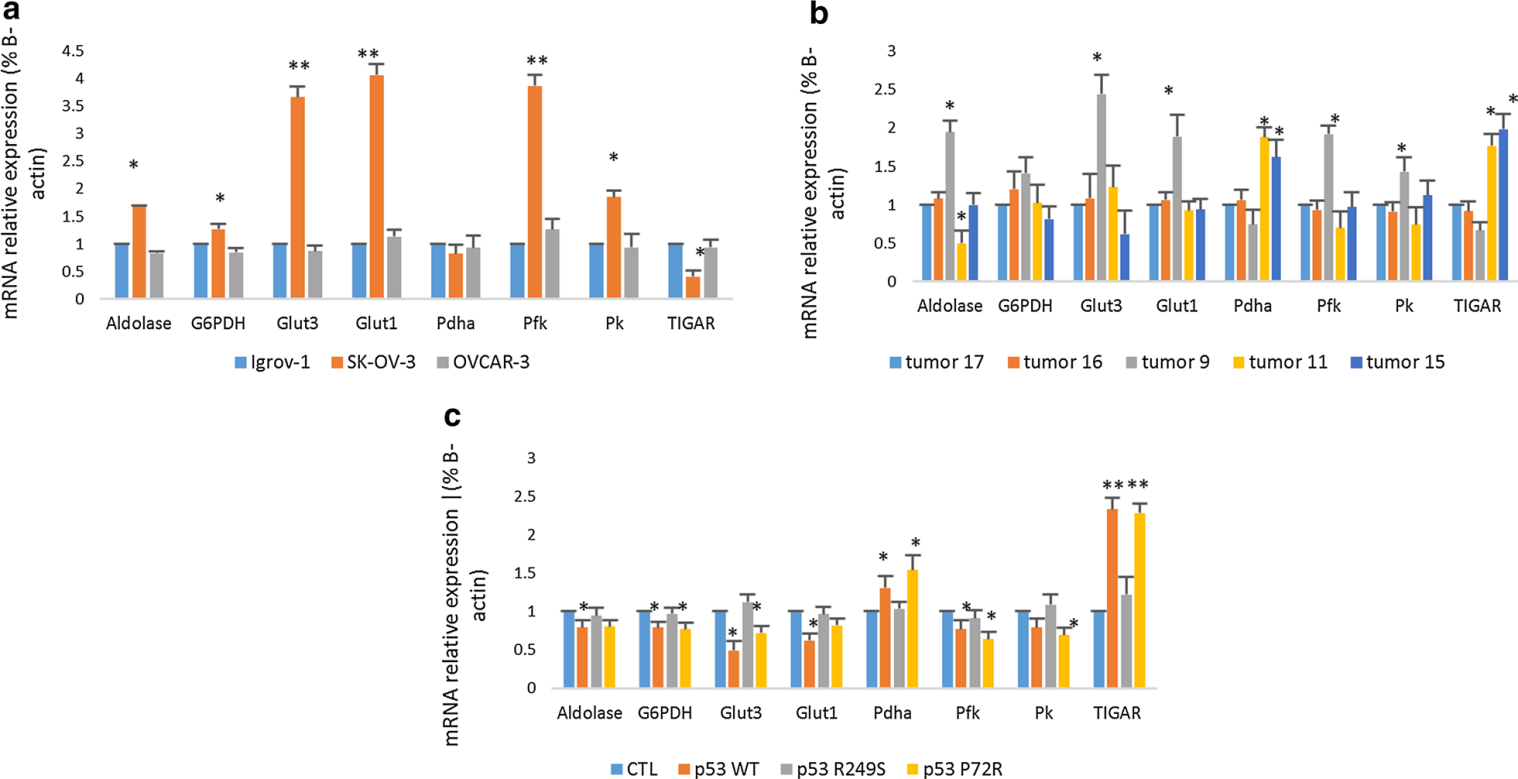
Effect of the domain mutations on the expression levels of p53 regulated glycolytic enzymes. qPCR for the p53 downregulated glycolytic enzymes aldolase, G6PDH, GLUT1 and 3, PFK and PK, and the p53 upregulated proteins PDHa and TIGAR was performed using the cDNA of the 3 ovarian cancer cell lines (**a**), the tumor cultured cells (**b**) and the transfected SK-OV-3 cells (**c**). Each value represents the mean of 3 assays. The control was set to 1, and the values are compared to the control. The results are displayed as the mean ± SD. *P < 0.05 versus the control, and **P < 0.01 versus the control

To investigate the reason behind these metabolism alterations, we studied the expression levels of the p53 downregulated enzymes GLUT 1 and 3, G6PD, aldolase, PFK and PK and the p53 upregulated enzymes PDHa and TIGAR. The results in Fig. 5 display a significant increase in the downregulated enzymes in the absence of p53 in the SK-OV-3 cells (Fig. 5a). A similar pattern was observed for tumor 9, which was caused mainly by the core domain mutation of p53 in the tumor 9 cells (Fig. 5b). An opposing pattern is observed for TIGAR and PDHa in both types of cells. Tumors 11 and 15 showed an increase in the expression of both PDHa and TIGAR, which can be explained by the fact that the overexpression of the protein that leads to the activation of the transcription of these two genes.

The same experiment was performed on the SK-OV-3 transfected cells, and Fig. 5c shows a significant effect on the expression of these enzymes when WT p53 and mutant p53 P72R were transfected into the cells. In fact, an increase was observed in the expression levels of p53 upregulated glycolytic enzymes, whereas the levels of downregulated enzymes decreased significantly. No effect was detected in the presence of the DNA binding domain mutant p53.

## Discussion

TP53 is a highly mutated tumor suppressor gene in human cancers and is known for its regulation of cell cycle and apoptosis initiation [18]. Clinically, mutant p53 is used to predict chemoresistance in some types of cancer. In fact, mutant p53 gain-of-function leads to the development of various chemoresistance mechanisms, such as DNA repair upregulation, enhanced drug efflux and metabolism, and apoptosis inhibition [19]. In both early and advanced stage serous ovarian cancers, TP53 mutations are detected in 60–70% of the cases [20, 21]. These mutations increase the risk of metastasis [22], correlate with resistance to platinum-based chemotherapy [7] and decrease the overall survival rates and progression-free survival [23]. Knowing that approximately 80% of the mutations are clustered in the region coding for the highly conserved DNA binding domain of the gene (exon 5–8), a majority of the hotspot mutants studied, such as R175H, R248W, R249S and R273C, belong to this specific region [24]. Interestingly, one hotspot mutation, affecting exon 4 of the TP53 gene, contributes to an increased risk of diabetes, obesity and metabolic dysfunction [25]. Combining these facts together, we investigated the prevalence and location of TP53 mutations and the effect of two hotspot mutations, P72R and R249S, on the protein’s activity as a transcription factor, apoptosis activator and glucose metabolism regulator. Our study analyzed a cohort of 30 ovarian carcinomas exhibiting TP53 sequence alterations and considered two main issues related to p53, including the expression of the protein by immunohistochemistry on paraffin-embedded tissues and the frequency of the mutations throughout the entire coding region of the gene by PCR-SSCP and sequencing. In our study, the proline rich domain, and more specifically exon 4, exhibited a higher mutation percentage (69%), which does not correlate with previous finding. In fact, a majority of the studies dealing with TP53 mutations restricted their investigations to the core domain of the gene, which might cause a misinterpretation of the results in the case of undetected mutations in other regions of the gene. The overexpression of p53, detected by IHC and qPCR, was mainly caused by TP53 mutations, which correlates with the results obtained by Yemelyanova et al, stating that the immunohistochemical pattern of p53 in tumors is a predictor of TP53 mutations [26].

P53 is the key regulator of the transcription of various genes. For example, P21 gene expression is directly activated by p53 by its recruitment to the promoter region [27]. MDM2 is also linked to p53 by an auto-regulatory negative feedback loop [28]. In this study, we investigated the effect of the two p53 mutants on the protein’s transcriptional activity by quantifying the expression of p21 and MDM2. The results obtained clearly showed a downregulation of the expression of both genes when the mutation affected the core domain of the protein. In fact, the R249S mutant protein undergoes distortion in its DNA binding domain [29], which may prevent it from binding to its appropriate site in the promoter region. The increase in the expression of p21 and MDM2 observed in the tumors with the proline rich domain mutation might be caused by the overexpression of p53. In fact, since the alteration does not affect the core domain of the protein, its transcriptional activity might be unaltered, which explains the observed overexpression of both of the target genes.

Despite its activity as gene expression regulator, p53 plays a key role in apoptosis initiation. To study the effect of both mutations on the ability of the protein to affect cellular viability and apoptosis induction, the p53-null Sk-Ov-3 cell line was transfected with both p53 mutants. The results clearly show a decrease in the viability of the transfected cells after 48 and 72 h of transfection, but there was no significant difference between the mutant types. Surprisingly, the mutation affecting codon 72 located in the proline rich domain did not contribute to a significant decrease in the apoptotic activity of p53. This may be explained by the results obtained by Thomas et al, stating that mutant P72R p53 is structurally wild type and exhibits the same affinity for a wide range of p53 DNA recognition sequences [30].

Recent studies have investigated the role of the p53 protein in the regulation of glucose metabolism. In fact, in the presence of WTp53, glucose transport decreases through the plasma membrane, and the expression of some glycolytic enzymes drops, which leads to a reduction in glucose consumption [31]. In addition, knowing that most of the pyruvate produced by glycolysis is converted into lactate instead of undergoing oxidation by the mitochondria in cancer cells, glucose consumption and lactate production vary proportionally [32]. Our results show a significant increase in glucose consumption and lactate production by the Sk-Ov-3 cells, and this may be due to the absence of p53 expression. The slight decrease in both concentrations observed in tumor 11 and 15 with the exon 4 mutation may be caused by the overexpression of the p53 protein, and in contrary, tumor 9 exhibited an increase in glucose and lactate concentration, which is probably caused by the mutation in the DNA binding domain of the p53 protein. The same aspect was observed when the Sk-Ov-3 cells were transfected with the three forms of p53. In fact, the decrease observed in both WT and P72R p53 might be triggered by the functional core domain of the protein, which inhibits the enzymes in the glycolytic pathway. To investigate the main reason behind this metabolic change, we investigated the effect of WT and mutant p53 on the expression of some glycolytic enzymes. The results displayed a significant increase in the p53-downregulated enzymes in the cell lines and tumors with an absent or DNA binding domain mutant p53, which was mainly caused by the lack of p53 activity in both cases. In contrast, the transfection of the cells with WT or P72R p53 produced an increase in the p53-upregulated enzymes, such as TIGAR and PDHa, which are the main inhibitors of glycolysis regulated by p53. This upregulation, coupled with the decreased glucose transporter expression, explains the decrease observed in glucose consumption and lactate production in these cells. These results might suggest that patients exhibiting the P72R mutation are less prone to develop metastasis due to the lower lactate production levels, which could lead to a better prognosis.

## Conclusion

In conclusion, our study demonstrates, for the first time, that p53 overexpression in ovarian cancer can be interpreted differently depending on the locus of the mutation affecting TP53. In fact, although P72R mutant exhibits the same features as WTp53 in its transcriptional activity, apoptosis induction ability and glucose metabolism regulation, this type of mutation contributes to p53 overexpression in tumor cells. Knowing that this overexpression is a marker of poor prognosis for ovarian cancer, the locus of the mutation causing this overexpression should be further investigated for proper interpretation of the routine immunohistochemistry results performed on ovarian cancer biopsies.

## Additional files

**Additional file 1: Table S1.** Sequence of primers.

**Additional file 2: Table S2.** Clinical and histopathological characteristics of tumors.
