# Supplementary material for: Different TP53 mutants in p53 overexpressed epithelial ovarian carcinoma can be associated both with altered and unaltered glycolytic and apoptotic profiles
Source: Cancer Cell Int. 2018 Jan 30;18:14. doi: 10.1186/s12935-018-0514-2 (PMC5791177; doi:10.1186/s12935-018-0514-2)
Supplement: Supplementary file 1 — Additional file 1: Table S1. Sequence of primers. [file 12935_2018_514_MOESM1_ESM.docx]

| **Gene** | **Forward Primer** | **Reverse Primer** |
| --- | --- | --- |
| ß-actin | 5-CATTCCAAATATGAGATGCGTTGT-3’ | 5’-TGTGGACTTGGGAGAGGACT-3’ |
| GAPDH | 5'-TCATCATCTCTGCCCCCTCT-3' | 5'-TCCGACGCCTGCTTCACCAC-3’ |
| P21 | 5’- AAGACCATGTGGACCTGT-3’ | 5’- GGTAGAAATCTGTCATGCTG-3’ |
| MDM2 | 5’- TGTAAGTGAACATTCAGGTG-3’ | 5’- TTCCAATAGTCAGCTAAGGA-3’ |
| Aldolase | 5’- TCAACCACACTCCGTCCACG-3’ | 5’- GTAGCAAGTTCCGGTGCTTC-3’ |
| G6PDH | 5’- TGAGCCAGATAGGCTGGAA-3’ | 5’- TAACGCAGGCGATGTTGTC-3’ |
| GLUT-3 | 5'-CCCAGATCTTTGGTCTGGAA-3' | 5'-AAGGGCTGCACTTTGTAGGA-3' |
| GLUT-1 | 5'-GATGATGCGGGAGAAGAAGG-3' | 5'-AAGACAGCGTTGATGCCAGAC-3' |
| PDHa | 5’- TTCTCAGAAGCCGGCAAGC -3’ | 5’- AGCACTGTTGTGACAGGAGG -3’ |
| PFK | 5'-ACTGACGCCTGTCGCTTATG-3' | 5'-GAGCGGGTTAGGTCCCTTCT-3' |
| PK | 5’-AGAACATCCTGTGGCTGGAC-3’ | 5’-ACCTTTCTGCTTCACCTGGA-3’ |
| TIGAR | 5’- CTGACTGAAACTCGCTAAGG-3’ | 5’- CAGAACTAGCAGAGGAGAGA-3’ |
| TP53 | 5’-AGATAGCGATGGTCTGGC-3’ | 5’-TTGGGCAGTGCTCGCTTAGT-3’ |
| TP53 exon 4 | 5’-CTGCTCCCCGCGTGGCCCCT-3’ | 5’-AGGGGCCACGCGGGGAGCAG-3’ |
| TP53 exon4 P72R | 5’-CGATATTGAACAATGGTTCA-3’ | 5’-TTTTCTGGGAAGGGACAGAA-3’ |
| Exon 2 | 5’-CAGGGTTGGAAGCGTCTCAT-3’ | 5’-CTTCCCACAGGTCTCTGCTA-3’ |
| Exon 3 | 5’-TAGCAGAGACCTGTGGGAAGC- 3’ | 5’-AGAGCAGTCAGAGGACCAGGT-3’ |
| Exon 4 | 5’-CGTTCTGGTAAGGACAAGGGT-3’ | 5’-AAGAAATGCAGGGGGATACGG- 3’ |
| Exon 5 | 5’-CTGTTCACTTGTGCCCTGAC-3’ | 5’-AACCAGCCCTGTCGTCTCTC-3’ |
| Exon 6 | 5’-GCTGGAGAGACGACAGGGCT-3’ | 5’-CAACCACCCTTAACCCCTCC- 3’ |
| Exon 7 | 5’-CTTGCCACAGGTCTCCCCAA-3’ | 5’-AGGGGTCAGCGGCAAGCAGA-3’ |
| Exon8 | 5’-TTCCTTACTGCCTCTTGCTT-3’ | 5’-AGGCATAACTGCACCCTTGG- 3’ |
| Exon 9 | 5’-AGCAAGCAGGACAAGAAGCG- 3’ | 5’-GCAAATGCCCCAATTGCAGG-3’ |
| Exon 10 | 5’-CGATGTTGCTTTTGATCCGTCA-3’ | 5’-ATCCTATGGCTTTCCAACCTAG-3’ |
| Exon 11 | 5’-TCCCGTTGTCCCAGCCTTAG- 3’ | 5’-TGGTATGTCCTACTCCCCATC-3’ |

**Table S1:** Sequence of primers
