# Supplementary material for: Different TP53 mutants in p53 overexpressed epithelial ovarian carcinoma can be associated both with altered and unaltered glycolytic and apoptotic profiles
Source: Cancer Cell Int. 2018 Jan 30;18:14. doi: 10.1186/s12935-018-0514-2 (PMC5791177; doi:10.1186/s12935-018-0514-2)
Supplement: Supplementary file 2 — Additional file 2: Table S2. Clinical and histopathological characteristics of tumors. [file 12935_2018_514_MOESM2_ESM.docx]

|  | **p53 mutations analysis** | | **p53 protein expression** | |
| --- | --- | --- | --- | --- |
| **Tumor characteristics** | p53 mutated | p53 wild type | overexpression | normal expression |
| **Patients number (%)** | 13 (43.3) | 17 (56.7) | 15 (50) | 15 (50) |
| **Median age (yr)** | 56.3 | 55.8 | 55.2 | 55.4 |
| **Histological subtype (%)**  **Serous**  **Mucinous**  **Endometrioid**  **Undifferentiated** | 9 (69.2)  -  3 (23.1)  1 (7.7) | 9 (52.9)  2 (11.7)  3 (17.7)  3 (17.7) | 10 (66.7)  -  3 (20)  2 (13.3) | 8 (53.4)  2 (13.3)  3 (20)  2 (13.3) |
| **Clinical stage (%)**  **II**  **III**  **IV** | 2 (15.4)  4 (30.7)  7 (53.9) | 1 (5.9)  5 (29.4)  11 (64.7) | 2 (13.3)  6 (40)  7 (46.7) | 1 (6.7)  4 (26.6)  10 (66.7) |
| **Grade (%)**  **I**  **II**  **III** | 1 (7.7)  3 (23.1)  9 (69.2) | 1 (5.9)  3 (17.7)  13 (76.4) | 1 (6.7)  4 (26.6)  10 (66.7) | 12 (80)  2 (13.3)  1 (6.7) |

**Table S2:** Clinical and histopathological characteristics of tumors.
